# A Modified Iterative Automatic Method for Characterization at Shear Resonance: Case Study of Ba_0.85_Ca_0.15_Ti_0.90_Zr_0.10_O_3_ Eco-Piezoceramics

**DOI:** 10.3390/ma13071666

**Published:** 2020-04-03

**Authors:** Lorena Pardo, Armando Reyes-Montero, Álvaro García, Alfredo Jacas-Rodríguez, Pilar Ochoa, Amador M. González, Francisco J. Jiménez, Manuel Vázquez-Rodríguez, María E. Villafuerte-Castrejón

**Affiliations:** 1 Instituto de Ciencia de Materiales de Madrid (ICMM), CSIC. c/Sor Juana Inés de la Cruz, 3, 24049 Madrid, Spain; alvarog@icmm.csic.es (Á.G.); ajacas@icmm.csic.es (A.J.-R.); 2 Departamento de Ingeniería de Procesos e Hidráulica, Universidad Autónoma Metropolitana-Iztapalapa, A.P. 55-534, CDMX, Mexico; ingaremo@gmail.com; 3 Departmento de Electrónica Física, Ingeniería Eléctrica y Física Aplicada, Universidad Politécnica de Madrid (UPM), c/Nikola Tesla, s/n, 28031 Madrid, Spain; pilar.ochoa@upm.es (P.O.); amador.m.gonzalez@upm.es (A.M.G.); franciscojavier.jimenez@upm.es (F.J.J.); 4 Departmento de Ingeniería Telemática y Electrónica (DTE), Universidad Politécnica de Madrid (UPM), c/Nikola Tesla, s/n, 28031 Madrid, Spain; m.vazquez@upm.es; 5 Instituto de Investigaciones en Materiales, Universidad Nacional Autónoma de México, A.P. 70360 Cd. Universitaria, C.P. 04510, CDMX, Mexico; mevc@unam.mx

**Keywords:** piezoelectrics, ferroelectrics, dielectrics, barium titanate, zirconates, ceramics, characterization, impedance, losses, shear

## Abstract

Coupling between electrically excited electromechanical resonances of piezoelectric ceramics is undesirable for the purpose of their characterization, since the material models correspond to monomodal resonances. However, coupling takes place quite often and it is unavoidable at the shear resonance of standard in-plane poled and thickness-excited rectangular plates. The piezoelectric coefficient *e*_15_, the elastic compliance *s*_55_^*E*^ and the dielectric permittivity component *ε*_11_^*S*^ for a piezoelectric ceramic can be determined, including all losses, using the automatic iterative method of analysis of the complex impedance curves for the shear mode of an appropriated resonator. This is the non-standard, thickness-poled and longitudinally excited, shear plate. In this paper, the automatic iterative method is modified. The purpose is to be able to deal with the analysis of the impedance curves of the non-standard plate as the periodic phenomena of coupling and decoupling of the main shear resonance and other resonances takes place. This happens when the thickness of the plate is reduced, and its aspect ratio (width of the excitation (*w*)/thickness for poling (*t*)) is increased. In this process, the frequency of the shear resonance also increases and meets those of other plate modes periodically. We aim to obtain the best approach for the shear properties of near coupling and to reveal both their validity and the limitations of the thus-obtained information. Finally, we use a plate of a Ba_0.85_Ca_0.15_Ti_0.90_Zr_0.10_O_3_ eco-piezoceramic as a case study.

## 1. Introduction

Polarized ferroelectric polycrystals, also known as piezoelectric ceramics or piezoceramics, remain one of the most important classes of electroceramics, with applications as sensors, transducers, actuators or motors in numerous human activities (health, communications, industry, etc.) [1]. Starting from the seminal studies in the 1940s, this multidisciplinary area of research has focused on the efforts of solid state and applied physicists and chemists as much as on those of materials scientists and electrical and electronic engineers. Two of today’s most exciting challenges for these materials are in the area of eco-friendly compositions and processing routes [2] and in the area of applications for clean and renewable sources of energy [3]. Nowadays, the need for a replacement for soft lead titanate–zirconate (PZT), the leading commercial composition, by “lead-free” compositions, which, in Europe, started in the 2000s [4,5], is imperative due to the environmental concerns of society.

The characterization of piezoelectric ceramics in the linear range is commonly achieved by the resonance method [6], from analysis of complex impedance measurements at the electrically excited electromechanical resonances of ceramics with regular geometries. For this purpose, both precise and accurate values of complex impedance and frequency are needed, which requires an analysis of the monomodal resonances. Measurements are standardized [7,8] and such procedures are widely used in science and industry for comparisons of materials. Nevertheless, standard procedures have a number of limitations when coefficients are used for modeling these materials by numerical methods. Specifically, accurate modeling of piezoelectric ceramics by Finite Element Analysis (FEA) requires the full matrix of material coefficients, which in turn needs to determine 10 coefficients, including electromechanical, dielectric and elastic losses. With this aim, methods of analysis of impedance curves have been developed [6], as an alternative to standard procedures, and still are a focus of research [9].

**Figure 1:**
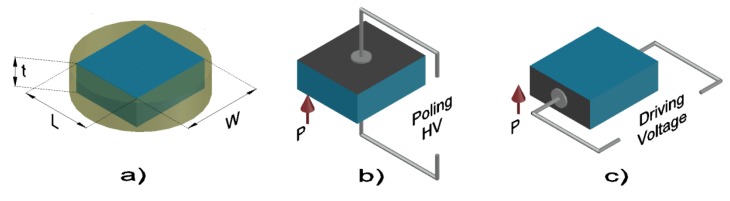
(**a**) The original ceramic disk; (**b**) the shear plate cut from the disk and then thickness (*t*) poled (after poling electrodes were removed and new ones attached); (**c**) the shear plate excited in the length between electrodes (*w*) of area *t*x*L*.

The shear mode and other natural modes of resonance of the standard in-plane polarized and thickness-excited rectangular ceramic plate are coupled [10,11,12]. This is an unavoidable phenomenon for this resonator type that leads to underestimation of the electromechanical shear coefficients [13,14]. To overcome this problem, a non-standard thickness-polarized length-excited shear plate was studied [15,16]. This resonator is as easy to pole as a thin disk and can be extracted from it (Figure 1). Besides, the thickness of the poled ceramic item can be reduced by careful polishing of the major faces. It is possible in this way to increase the aspect ratio of this resonator and efficiently increase the frequency of the shear mode. This reduces the coupling of the thickness-driven shear mode and lateral modes (and their overtones) taking place periodically.

The shear properties were accurately determined for a commercial PZT [16] and for eco-piezoceramics with lead-free composition Ba_1−x_Ca_x_Ti_0.90_Zr_0·10_O_3_ (BCZT), where x = 0.10–0.18 [17]. To this aim, the automatic iterative method of analysis of the complex impedance spectra at the resonance [18,19,20] of such a non-standard shear plate was implemented. Since the decoupling of the fundamental shear mode and other undesired natural resonance modes of the non-standard plate can be effectively achieved, the required spectrum to analyze was obtained. Indeed, a number of uncoupled shear resonances of the plate, amenable for material characterization, including all losses, were effectively obtained in the range of the aspect ratios (length for excitation (*w*)/thickness for poling (*t*)) between nine and 15 for PZT and 5.5 and 12.5 for BCZT. It must be noted that the standard measurement methods recommend to use plates with *w*/*t* and *L*/*t* > 20 [10] and *w*/*t* and *L*/*t* > 32 [8], to minimize the effect of the undesired modes on the shear mode.

The coupling of modes is undesirable, but takes place quite often when studying piezoelectric ceramics, e.g., when a new system of solid solutions is addressed and a broad number of compositions must be characterized. Uncoupled planar modes of thin disks, thickness poled, which are easy to obtain, are often selected for this purpose. This has the drawback of providing only partial knowledge about the material, which possesses a well-known anisotropy [7,8,21,22]. Methods to deal with the analysis of some impedance curves of coupled resonances could reveal both the value and the limitations of the thus-obtained information. The automatic iterative method was here modified to allow expert analysis of the coupled resonance of thickness-poled and longitudinally excited non-standard shear piezoelectric ceramic plates. We aim to obtain the best approach for the calculated shear properties of the material near coupling. In this work, as a case study and due to the interest in the composition and scarce shear data on this solid solution system [17,23], the resonance spectra of a plate of Ba_0.85_Ca_0.15_Ti_0.90_Zr_0.10_O_3_ eco-piezoceramic are analyzed as the coupling of shear and lateral modes evolves with the change in the aspect ratio (*w*/*t*) of the plate. The evolution of the spectrum (*R* and *G* peaks) of the shear mode of the non-standard shear ceramic item was studied by measuring resonators of different aspect ratios and obtained by the progressive reduction in their thickness.

## 2. Material and Methods 

### 2.1. Material

A rectangular plate of Ba_0.85_Ca_0.15_Ti_0.90_Zr_0.10_O_3_ piezoceramic, with an initial thickness for polarization *t* (distance between electrodes for poling) = 1.09 mm, lateral dimensions of *L* = 8.18 mm and *w* (distance between electrodes for electrical excitation of the resonance) = 6.20 mm and a density of 5.62 g.cm^−3^, was fabricated by conventional solid state route, as explained elsewhere [24], by sintering at 1400 °C for 2 h in air. Silver paint electrodes of area *L* × *w* were painted on both major faces and annealed at 600 °C for 30 min. The ceramics were thickness-poled at room temperature under 3 kV.mm^−1^ for 30 min. After poling, the electrodes were removed by fine polishing and new electrodes of area *L* × *t* were attached for the longitudinal electrical excitation of the electromechanical resonances and for the impedance measurements. Thickness was reduced in steps of 0.01 mm to a final value of *t* = 0.5 mm, in order to change the aspect ratio (*w*/*t*), and the impedance spectrum was measured again at each step.

### 2.2. The Automatic Iterative Method for Analysis of Impedance Curves

The complex impedance was measured using an impedance analyzer (model HP4192A-LF impedance analyzer, Hewlett-Packard, Palo Alto, CA, USA). The complex piezoelectric coefficient *e*_15_ and the complex elastic compliance *s*_55_^*E*^, as well as the complex dielectric permittivity at the resonance frequency (*ε*_11_^*S*^), were directly calculated using the automatic iterative method of analysis of the impedance measurements [16,18] and were previously reported [17]. They will be considered in this work for the sake of comparison with the results here obtained using the modified method.

**Figure 2:**
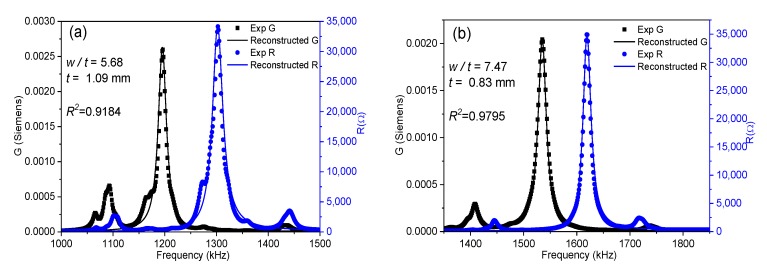
Impedance spectra for the shear mode of the Ba_1−x_Ca_x_Ti_0.90_Zr_0·10_O_3_ (BCZT) plate for aspect ratios (**a**) *w*/*t* = 5.68; (**b**) *w*/*t* = 7.47. Measured *G* and *R* peaks (symbols) and reconstructed peaks (solid lines) are displayed.

Commonly, Z* is plotted as the modulus and phase angle; instead, here we use an equivalent representation. The peaks of the real part of Z*, the resistance (*R*), and the real part of the complex admittance, the inverse of the impedance (Y* = 1/Z*), the conductance (*G*), give a convenient plot for the use of the automatic iterative method (Figure 2) [6]. The frequencies of the maximum *G* and *R* values, *fs* and *fp*, respectively, are two of the four frequencies of interest, which are automatically determined by the software. The other two frequencies, *f*_1_ and *f*_2_, are those of maximum piezoelectric energy and are also automatically calculated at each iteration [18]. Experimental *G* and *R* peaks (symbols) for the BCZT plate are shown in Figure 1 together with the reconstructed spectra (continuous lines), which was obtained after the material parameters were determined. The software computes the shear electromechanical coupling factor, *k*_15_, from [7]:

(1) $$d_{15}=k_{15}{\left(\varepsilon_{11}{}^{T}s_{55}{}^{E}\right)}^{1/2}$$

where

(2) $$e_{15}=h_{15}\varepsilon_{11}{}^{S},d_{15}=e_{15}s_{55}{}^{E}$$

and

(3) $$\varepsilon_{11}{}^{T}=\frac{\varepsilon_{11}{}^{S}}{1-h_{15}{}^{2}s_{55}{}^{D}\varepsilon_{11}{}^{S}}$$

**Figure 3:**
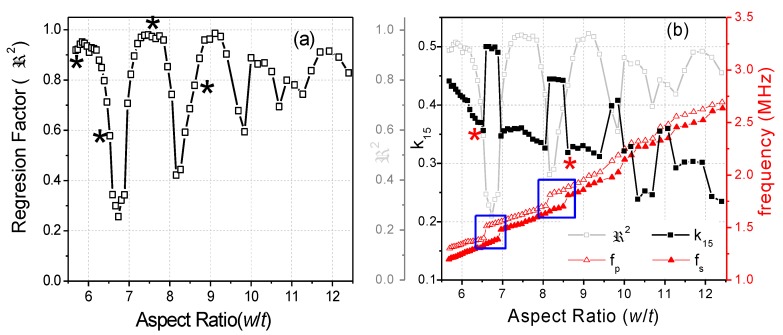
Evolution as a function of the aspect ratio of the BCZT plate: (**a**) of the reconstructed to the experimental resonance spectra regression factor, ${ℜ}^{2}$, (the points marked with (*) correspond to the spectra of Figure 2, which are examples of uncoupled shear modes with high ${ℜ}^{2}$, and Figure 4, examples of coupled modes with low ${ℜ}^{2}$) and (**b**) of the frequencies *fp* and *fs* and the electromechanical coupling factor, *k*_15_; squares indicate anomalies in the difference (*fp* − *fs*).

**Figure 4:**
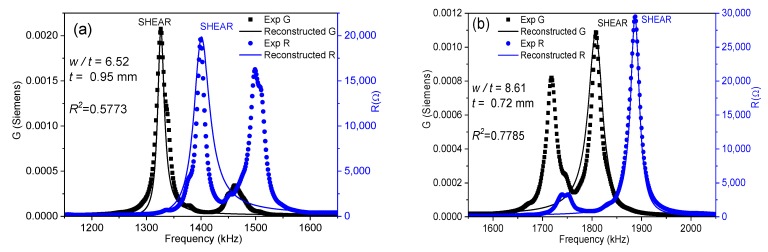
Impedance spectra showing coupled modes (low ${ℜ}^{2}$) for the BCZT plate for aspect ratios (**a**) *w*/*t* = 6.52; (**b**) *w*/*t* = 8.61. Experimental *G* and *R* peaks (symbols) are displayed, as well as reconstructed peaks (solid lines).

The reconstruction of the spectra is carried out by the calculation of the complex admittance as a function of the frequency by means of the analytical expression of the resonance that was just solved iteratively to obtain the coefficients from the impedance values. For the resonance mode under study, the expression used is:

(4) $$Y=G+iB=i\frac{2\pi ftL\varepsilon_{11}^{s}}{w}+i\frac{2te_{15}^{2}}{w}\sqrt{\frac{s_{55}^{E}}{\rho }}\tan\left(\pi ft\sqrt{\rho s_{55}^{E}}\right)$$

where *ρ* is the ceramic density. This equation is valid when *L*, *w* >> *t* [15]. In each iteration, the software solves by a numerical method a system of four equations, like Equation (4), for the values of *Y* measured at the four mentioned frequencies [18] until a convergence criterion of the determined coefficients is met. The residuals for these *R* and *G* curves (a residual being the difference between the experimental value for a given frequency and the value of the reconstructed spectrum at the same frequency) gives us a regression factor for the iterative analysis (${ℜ}^{2}$). This parameter accounts for the ability of Equation (4) and these coefficients to characterize the material at this mode of resonance. The higher the coupling of the main shear resonance with other, undesired, resonances, the lower the ${ℜ}^{2}$ value (Figure 2a). The closer the experimental resonance to a monomodal resonance corresponding to the analytical expression in Equation (4), the closer the ${ℜ}^{2}$ value is to one (Figure 2b). For this reason, the previous report on shear coefficients of BCZT included only those calculated for the highest values of ${ℜ}^{2}$ [17]. Figure 3a shows the evolution of ${ℜ}^{2}$ as a function of the aspect ratio and Figure 3b compares this with the evolution of the shear electromechanical coupling factor (*k*_15_), *fs* and *fp*. Figure 4 shows the spectra for two aspect ratios with low ${ℜ}^{2}$ and high coupling, marked with (*) in Figure 3a.

Figure 3a shows the periodicity of the decoupling (Figure 2)–coupling (Figure 4) phenomenon of the main shear mode with the lateral modes. This happens because lateral resonances take place periodically and the shear mode moves continuously towards higher frequencies as the aspect ratio increases. Consequently, there are periodical coincidences in the frequencies of the two types of resonances. The electromechanical coupling factor accounts for the amount of the electrical to mechanical energy transfer in a given mode. Figure 3b shows that the highest values of *k*_15_ correspond to the lowest values of ${ℜ}^{2}$. This was also observed in the study of a PZT ceramic [16]. However, coupling produces a transfer of energy from the excited mode to the spurious one, with the consequence of a lower amount of energy transduction in the shear mode. Therefore, such high values of *k*_15_ lack physical meaning. Let us comment on the origin of this result as follows.

Figure 4 shows the spectra of two coupled modes. Their coupling coefficients are marked with (*) in Figure 3b. These spectra are characterized by double peaks, either for the *R* curve, Figure 4a, or for the *G* curve, Figure 4b. 

The *G* peak of the shear mode in Figure 4a is still well separated from that of the following lateral mode and, therefore, both peaks are distinguishable. The software of the iterative method detects correctly the *fs* of the shear mode, for the maximum value of *G*. The *R* peak of the shear mode in Figure 4a is altered by the close presence of a lateral mode at ≈1500 kHz, which is excited as well. As a consequence, both modes have *R* peaks of similar height. The maximum of *R* for the shear mode is still higher than the maximum of the *R* for the lateral mode. Therefore, *fp* is well detected. The corresponding *k*_15_ coupling coefficient (Figure 3b) is not much different than that measured for the previous aspect ratios and follows the trend of decreasing as the aspect ratio and the coupling between modes gets higher and, consequently, as the transfer of energy to the mechanical vibration of the spurious mode takes place. 

Similarly, for the spectrum of Figure 4b, both characteristic frequencies of the shear mode are well determined automatically by the software. Another lateral mode is excited at ≈1700 kHz. The maximum of *G* for the shear mode is also higher than the maximum of *G* for this lateral mode and *fs* is also correctly determined. It is clear, by the shape of the reconstructed spectra and the low values of ${ℜ}^{2}$, that the two spectra in Figure 4 are not optimal for the material characterization; however, the calculated coupling factors from them (marked with (*) in Figure 3b), though affected by coupling, are not absurd, as the frequencies needed for the iterative analysis can be automatically determined.

**Figure 5:**
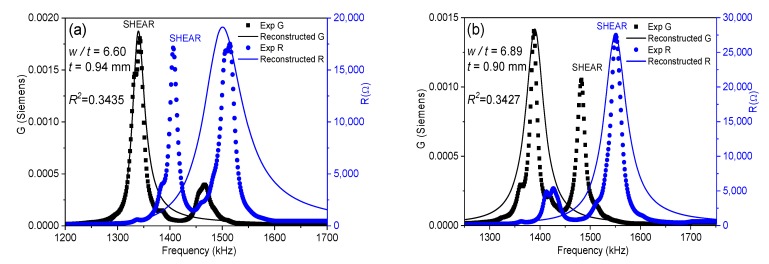
Impedance spectra showing coupled modes (very low ${ℜ}^{2}$) for the BCZT plate for aspect ratios (**a**) *w*/*t* = 6.60; (**b**) *w*/*t* = 6.89. Experimental *G* and *R* peaks (symbols) are displayed, as well as reconstructed peaks (solid lines).

In between the aspect ratios that result in spectra like those in Figure 4, e.g., at the first valleys of the ${ℜ}^{2}$ plot of Figure 3a, for 6.52 < *w*/*t* < 6.96 and 8.05 < *w*/*t* < 8.61, we measured the spectra as shown in Figure 5. The spectrum in Figure 5a is characterized by double peaks of *R*, when the shear frequency approaches the frequency of the lateral mode. The height of the *R* peak corresponding to the shear mode is lower than that corresponding to the lateral mode until the frequency of the shear resonance surpasses that of the lateral mode, as in Figure 5b. As the aspect ratio increases, the spectrum becomes like the one shown in Figure 5b, characterized by double peaks of *G*. At this point, the height of the *G* peak corresponding to the shear mode is lower than that corresponding to the lateral resonance. This leads to the incorrect automatic determination of the frequencies *fs* and *fp* and overestimation of the difference (*fp* − *fs*), as marked in Figure 3b. Consequently, the corresponding *k_15_* coupling coefficient is also overestimated and other coefficients also have anomalies [17].

The origin of the absurd values of *k*_15_ (Figure 3b) and other material parameters in the intervals of aspect ratio mentioned above is mainly a consequence of the incorrect selection of the frequencies for the shear mode used to solve Equation (4). This would also affect the calculation by any other method [7,8]. To avoid this, we here propose a modification of the analysis method.

### 2.3. The Modified Automatic Iterative Method

The determination of *fs* and *fp* for the modified automatic iterative method involves the expert choice of the shear resonance frequencies, based on the knowledge of the explained above evolution of the spectra, as the coupling of the shear and lateral modes takes place when the plate aspect ratio increases. This required measurements in resonators of different dimensions were obtained by the progressive reduction in the thickness of the non-standard ceramic plate, for proper mode identification. The modified method software allows the operator to graphically determine the *R* or *G*, or even both, maxima when double peaks are measured and, therefore, gives us the chance to select the proper frequency that will be used in the calculation using Equation (4) and the iterative procedure explained elsewhere [16,18].

**Figure 6:**
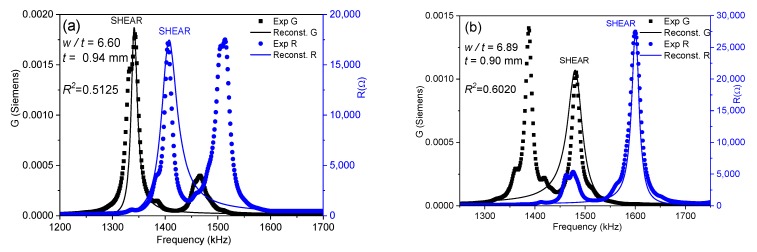
Impedance spectra showing coupled modes for the BCZT plate for aspect ratios (**a**) *w*/*t* = 6.60; (**b**) *w*/*t* = 6.89. Experimental *G* and *R* peaks (symbols), also shown in Figure 4, together with the reconstructed peaks using the material coefficients determined by the modified automatic iterative method (solid lines), are displayed.

Figure 6 shows the same spectra as Figure 5 compared with the reconstructed peaks after the parameter calculation with the modified method. The increase in ${ℜ}^{2}$ for both spectra in Figure 6 is apparent. 

**Figure 7:**
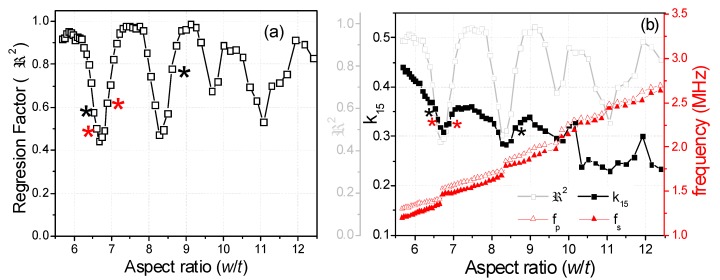
Evolution as a function of the aspect ratio of the BCZT plate: (**a**) of the regression factor, ${ℜ}^{2}$, reconstructed to the experimental shear resonance spectra (the points marked with (*) correspond to the spectra of Figure 4 and Figure 6, which are examples of coupled modes with low (black) and very low (red) ${ℜ}^{2}$, respectively) and (**b**) of the frequencies *fp* and *fs* and the electromechanical coupling factor, *k_15_*; all parameters of coupled modes were recalculated by the modified automatic iterative method.

Figure 7a shows the evolution of ${ℜ}^{2}$ as a function of the aspect ratio of the BCZT plate and Figure 7b compares this with the evolution of the calculated shear electromechanical coupling factor (*k*_15_), *fs* and *fp*. 

For Figure 7a,b, the analyses of similar spectra to those in Figure 5, corresponding to the valleys of the ${ℜ}^{2}$ plot in Figure 3a, were revised using the modified iterative method. As a result, the increase in the ${ℜ}^{2}$ in Figure 7a with respect to values in Figure 3a is also apparent. ${ℜ}^{2}$ never takes values below 0.44 in the revised calculations. 

Since ${ℜ}^{2}$ is used as criteria of validity of the method and of the calculated coefficients, the modified method increases the reliability of these coefficients across the studied periodic coupling–decoupling phenomenon. Furthermore, the absurd values of *k*_15_ are not observed in Figure 7b, where there is a quasi-parallel evolution of the two characteristic frequencies of the shear resonance, except in critical points corresponding to the “jump” of the frequencies of the shear mode over those of each lateral mode.

As already discussed regarding spectra in Figure 4, the spectra in Figure 6 are not optimum for the material characterization. However, the calculated parameters obtained from them by the modified automatic iterative method are not absurd. Therefore, in the following, we will show and discuss the calculated parameters obtained from the BCZT plate as the aspect ratio (*w*/*t*) increases, including those from coupled modes.

## 3. Results

### 3.1. Elastic Properties 

**Figure 8:**
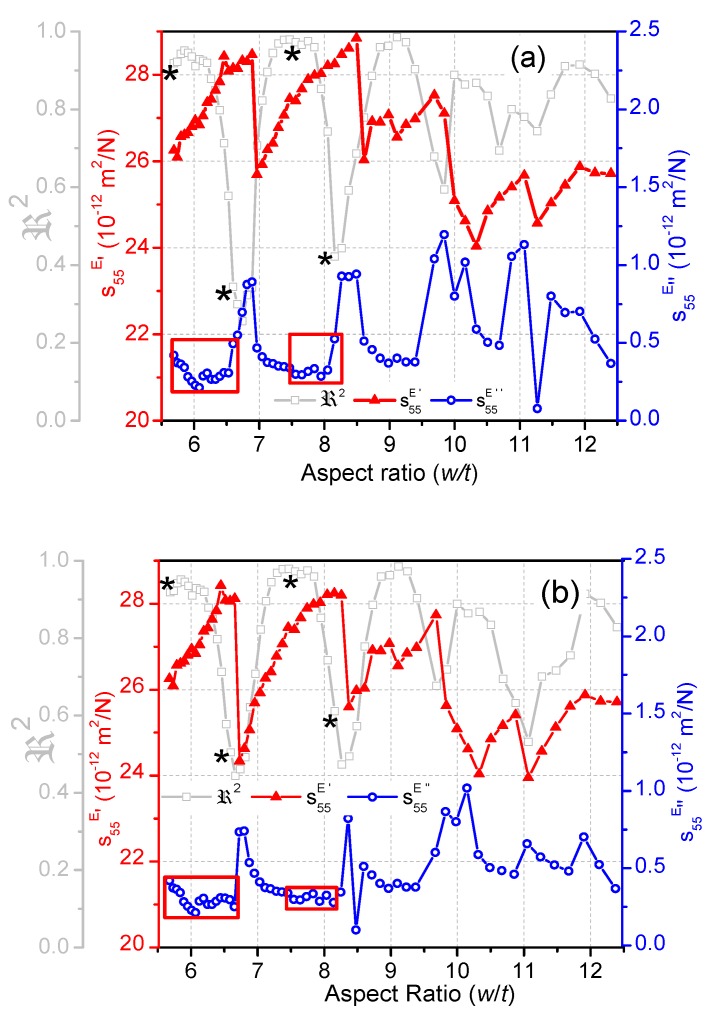
Evolution of the complex elastic compliance *s*_55_^*E*^ as a function of the aspect ratio of the BCZT plate; the real and the imaginary parts (*s*_55_^*E*^′ and *s*_55_^*E*^″) are both plotted for: (**a**) calculation by the automatic iterative method; (**b**) recalculation by the modified method. The evolution of ${ℜ}^{2}$ is shown as a reference.

Figure 8a,b show the evolution of the complex elastic constant calculated by the iterative method and the modified one, respectively. The elastic *s*_55_^*E*^ of the material depends mainly on the frequency of the resonance, as can be obtained from the following relationships [7]:

(5) $$s_{55}{}^{E}=\frac{1}{c_{55}{}^{E}},\;c_{55}{}^{E}=4(1-k_{15}{}^{2})\rho f_{p}{}^{2}t^{2}$$

Therefore, when the characteristic frequencies of the shear mode are correctly determined by the modified method, there is no artifact affecting the evolution of the calculated compliance *s*_55_^*E*^ with the aspect ratio (*w*/*t*). Abrupt jumps in the recalculated compliance (real part) from a maximum to a local minimum are shown in Figure 8b. These jumps take place in parallel to the jumps in the frequencies of the shear mode over those in each lateral mode, at the minima of the ${ℜ}^{2}$ periods. From each jump to the next, the recalculated compliance *s*_55_^*E*^ increases as the frequency of the shear mode increases. Since the material coefficient *s*_55_^*E*^′ is inversely proportional to the fp of the shear mode, this is the result of the overall modification of the spectrum near coupling (Figure 6), which limits the use of Equation (4). Unless we know if the aspect ratio of the measured plate is moving away from or coming closer to the nearest one for a coupled mode at ${ℜ}^{2}$ minima, it is not possible to know if the calculation is underestimating or overestimating *s*_55_^*E*^′. The deviation from the material coefficient, calculated at ${ℜ}^{2}$ maxima, can be estimated as ±4% by looking at the second period in Figure 8b (7 < *w*/*t* < 8). 

The recalculated imaginary part of the compliance using the modified method (Figure 8b) has fluctuations in narrower ranges of the aspect ratio than when calculated by the original method (Figure 8a). Fluctuations also take place at the minima of the ${ℜ}^{2}$ periods. By the use of the modified method, the dispersion in the first semi period, marked with red squares in Figure 8, decreases. Even so, for the best case and excluding the fluctuations, such a dispersion is of ±9% of the material coefficient.

### 3.2. Dielectric Properties

**Figure 9:**
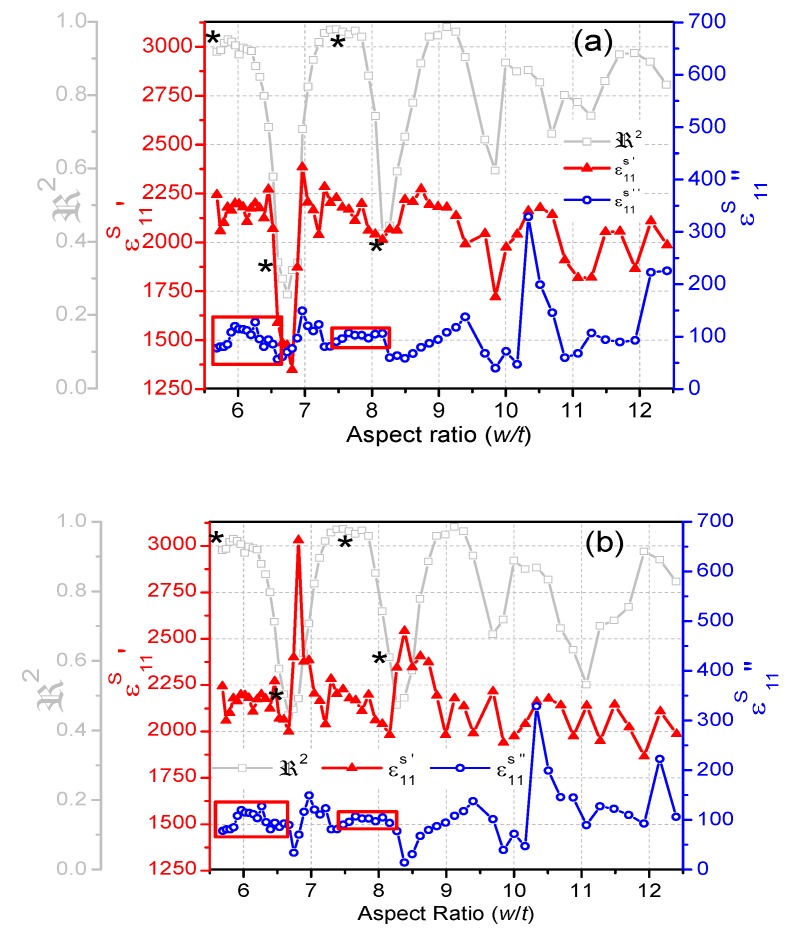
Evolution of the complex dielectric permittivity *ε*_11_^*S*^ as a function of the aspect ratio of the BCZT plate; the real and the imaginary parts (*ε*_11_^*S*^′ and *ε*_11_^*S*^″) are both plotted: (**a**) as-calculated by the automatic iterative method; (**b**) as-recalculated by the modified automatic iterative method. The evolution of ${ℜ}^{2}$ is shown as a reference.

The dielectric permittivity *ε*_11_^*S*^ obtained by the iterative method (Figure 9a) and the recalculated permittivity (Figure 9b) depend on the values of the admittance at the four frequencies used for the calculation. Therefore, the complex *ε*_11_^*S*^ is strongly affected by the coupling of modes that determines the shape of the spectrum near coupling (Figure 5). 

When the characteristic frequencies were not correctly determined, anomalously low values of the real part of the permittivity (Figure 9a) were calculated for spectra showing very low ${ℜ}^{2}$ (Figure 5). By using the modified method, we obtain a narrower range for the aspect ratio of the anomalies in the real part of *ε*_11_^*S*^ (Figure 9b), but still obtain a broad dispersion of the real part of the permittivity value, due to the changes in the shape of *R* and *G* curves in the shear mode, when dispersion takes place near the lateral modes (Figure 6). 

Though the trend is not so clearly seen as for *s*_55_^*E*^, jumps in the recalculated permittivity (real part) with anomalously large values (up to 40% higher than the permittivity of the material) can also be observed at the minima of the ${ℜ}^{2}$ periods (Figure 9b). From there until the next jump, the recalculated *ε*_11_^*S*^ slightly decreases as the frequency increases. 

The imaginary part of *ε*_11_^*S*^ shows anomalies with minimum values at the mentioned frequency jumps, meaning an increase in the dielectric loss tangent. As with the recalculated *s*_55_^*E*^″, the dispersion of *ε*_11_^*S*^″ in the first semi period, marked with red squares in Figure 9, decreases when recalculated. Even so, for the best case, the dispersion is of ±10% of the imaginary part of the material coefficient calculated for ${ℜ}^{2}$ maxima. 

### 3.3. Piezoelectric Properties

**Figure 10:**
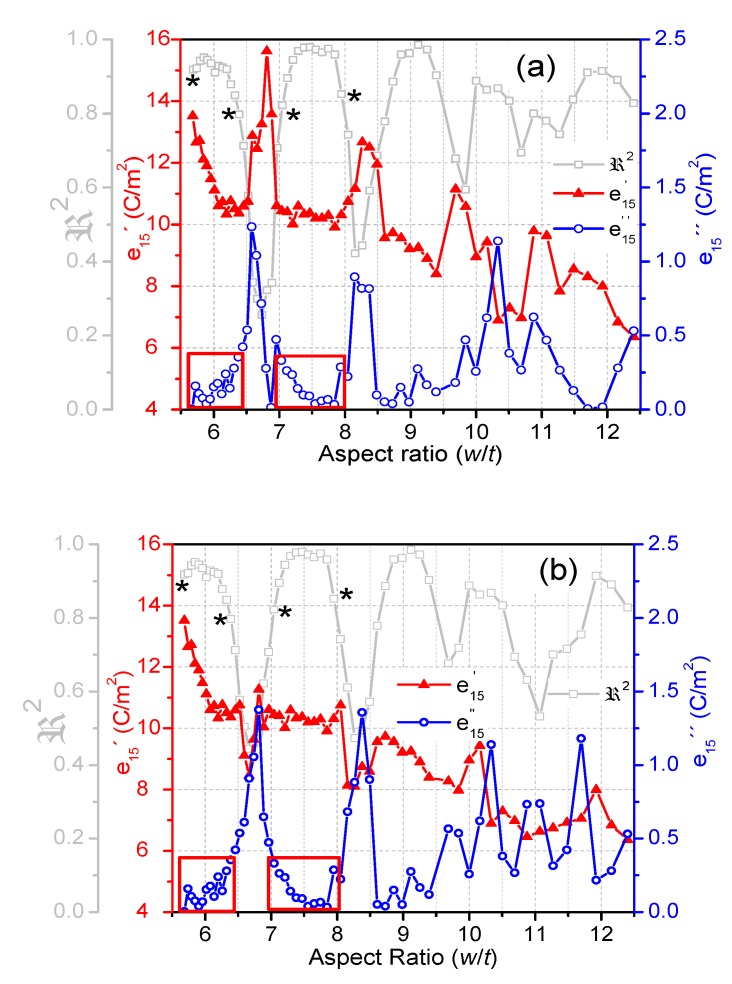
Evolution of the complex piezoelectric coefficient *e*_15_ as a function of the aspect ratio of the BCZT plate; the real and the imaginary parts (*e*_15_′ and *e*_15_″) are both plotted: (**a**) calculated by the automatic iterative method; (**b**) recalculated by the modified automatic iterative method. The evolution of ${ℜ}^{2}$ is shown as a reference.

The calculated piezoelectric coefficient *e*_15_′ by the automatic iterative method (Figure 10a), was overestimated due to the erroneous selection of the frequencies of the shear mode near the coupling with a lateral mode, similarly to what happened with the coupling factor (Figure 3b). Recent FEA modeling [25] shows that, under coupling, the mode of movement of the non-standard shear plate undergoes a dynamic clamping and the plate vibrates inhomogeneously. Therefore, the resonator is not amenable for characterization and those calculated coefficients for very low ${ℜ}^{2}$ lack physical meaning. Similar to the coupling factor (Figure 7b), after the recalculation with the modified method, *e*_15_′ exhibits minima in the aspect ratio with the highest coupling (Figure 10b) and lowest ${ℜ}^{2}$, which is meaningful. This also takes place in narrower ranges of *w*/*t*. Moreover, the deviation from the material coefficient of the recalculated *e*_15_′ is lower. Superimposed with this periodic evolution, in a similar manner to the coupling factor (Figure 3b), a continuous decrease in the recalculated *e*_15_′ as a function of the aspect ratio and frequency (Figure 10b) is observed. This decrease as w/t increases is characteristic of BCZT [17], and was not observed for PZT [16], which is out of the scope of this work.

The fluctuations in the calculated *e*_15_″ became wide peaks when recalculated by the modified method. These peaks are wider than for any other parameter analyzed here and, again, their maxima took place together with the ${ℜ}^{2}$ minima (Figure 10b). The deviations from the material *e*_15_″ are higher than for any of the other studied parameters. To consider only those calculations for ${ℜ}^{2}$ > 0.80, it is necessary to reduce the dispersion of *e*_15_″ to close to 100% of the material coefficient that is calculated for the maxima of ${ℜ}^{2}$. Consequently, a small deviation from this condition in the maxima of ${ℜ}^{2}$ is critical for the determination of the shear electromechanical losses of the material.

## 4. Conclusions

The automatic iterative method of analysis of the impedance curves at the shear electromechanical resonance of non-standard thickness-poled longitudinally excited piezoelectric ceramic rectangular plates was critically analyzed. Results on Ba_0.85_Ca_0.15_Ti_0.90_Zr_0.10_O_3_ plates were considered in this analysis, which required measurements on samples of different dimensions to properly identify the resonance modes. For this, impedance curves were obtained as a function of the increasing aspect ratio (length for excitation (*w*)/thickness for poling (*t*)), by reducing the plate thickness. It is concluded that the origin of the anomalies in the material parameters at the intervals of very low ${ℜ}^{2}$ is mainly a consequence of the incorrect selection of the frequencies for the shear mode used to solve Equation (4). This would also affect any other calculation based on such frequencies [7,8]. To avoid this, the iterative method was here modified to allow the expert analysis of shear modes when coupling with the periodic lateral modes takes place. The modified software allows graphically determining the *R* or *G*, or even both, values for the maxima when double peaks are measured and, therefore, gives the operator the choice of the proper frequencies that will be used in the calculation using Equation (4) and the iterative procedure.

Recalculated material parameters by the modified method confirmed previous experimental observations regarding the underestimation of the electromechanical properties, when the coupling of modes takes place, and the critical dependence on decoupling for the accurate determination of electromechanical losses. The periodic evolution of the elastic and dielectric shear coefficients recalculated by the modified iterative method as the aspect ratio of the plate increases was related to the periodicity of ${ℜ}^{2}$. The anomalies in real and imaginary parts of the coefficients take place at the points of the minima of ${ℜ}^{2}$ periods. Calculations with ${ℜ}^{2}$ > 0.80 (in between the points marked with (*) in the first cycles of Figure 10) are compulsory to get an estimate of the electromechanical losses. Low ${ℜ}^{2}$ characterized calculation from coupled modes and leads to deviations in the material coefficients.
